# Reply to Kang, “Distinct thermodynamic strategies in cello-oligosaccharide binding by *Anaerocellum bescii* substrate-binding proteins”

**DOI:** 10.1128/aem.00545-26

**Published:** 2026-06-17

**Authors:** Hansen Tjo, Virginia Jiang, Jerelle A. Joseph, Jonathan M. Conway

**Affiliations:** 1 Department of Chemical and Biological Engineering, Princeton University 214888, Princeton, New Jersey, USA; 2 Omenn-Darling Bioengineering Institute, Princeton University 6740, Princeton, New Jersey, USA; 3 Molecular Biology Department, Princeton University 6740, Princeton, New Jersey, USA; 4 Andlinger Center for Energy and the Environment, Princeton University 6740, Princeton, New Jersey, USA; 5 High Meadows Environmental Institute, Princeton University 460758, Princeton, New Jersey, USA; University of Nebraska-Lincoln, Lincoln, Nebraska, USA

## REPLY

We thank Jonghoon Kang for highlighting length-dependent enthalpy-entropy compensation (EEC) in Athe_0597 (1), based on the thermodynamic measurements reported in our original study (2). This prompted us to ask whether similar thermodynamic patterns are evident across other carbohydrate-binding SBPs in *A. bescii*.

**Fig 1:**
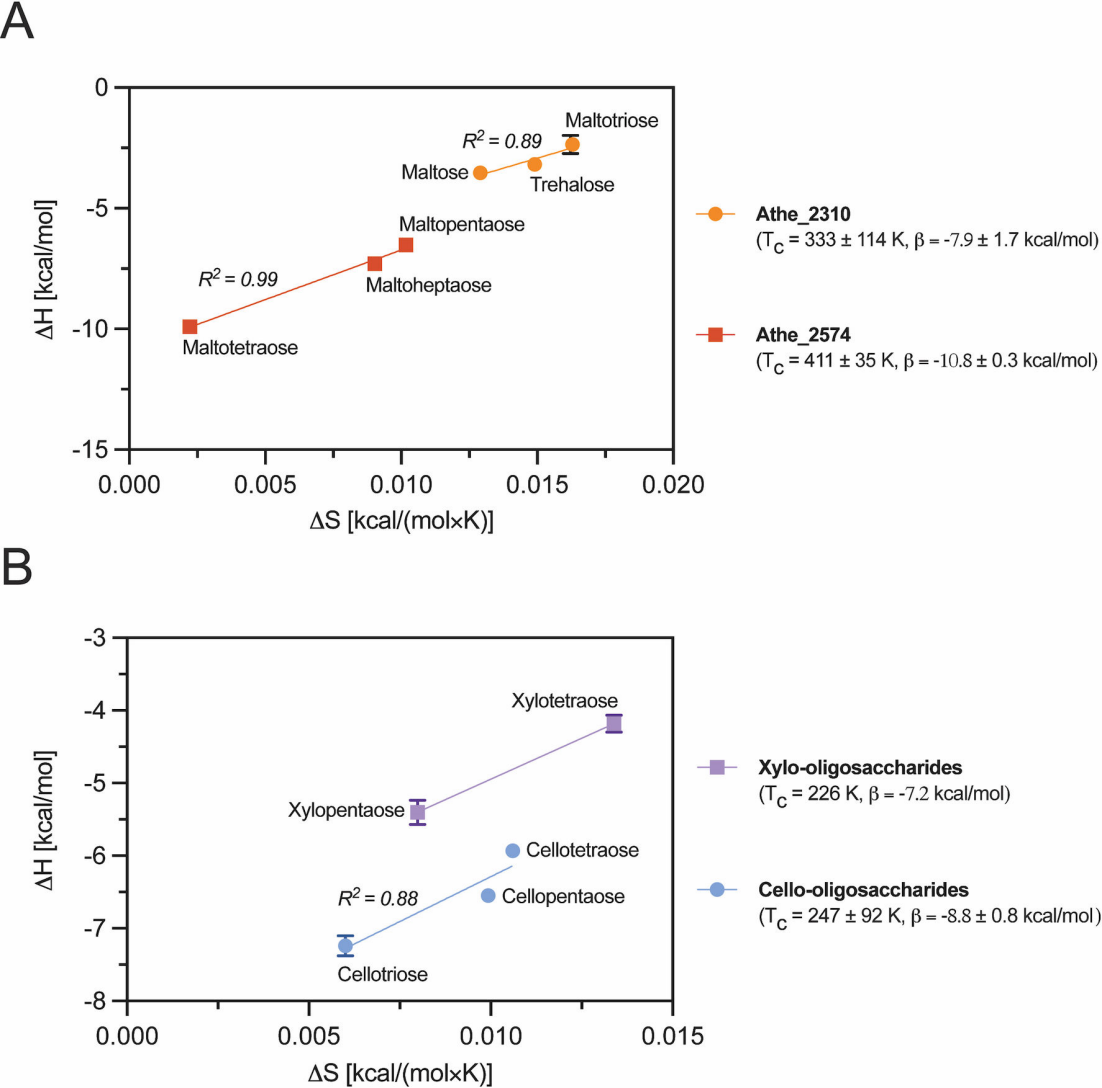
ABC substrate-binding proteins (SBPs) from *A. bescii* exhibit apparent EEC in oligosaccharide binding. (**A**) Binding of malto-oligosaccharides to two distinct SBPs. Athe_2310 and Athe_2574 each show an apparent linear relationship between binding enthalpy and entropy across malto-oligosaccharide ligands of varying lengths, consistent with length-dependent EEC behavior. (**B**) Binding of Athe_2052 to two distinct ligand classes. The multifunctional SBP Athe_2052 exhibits similar thermodynamic partitioning across two distinct ligand classes (β-1,4-linked xylo-oligosaccharides and β-1,4-linked cello-oligosaccharides), based on comparable fitted compensation temperatures (*T*_*c*_ = 226 K and 247 ± 92 K, respectively). Error bars represent the experimental error associated with the enthalpy change of binding as reported by the MicroCal PEAQ Isothermal Titration Calorimeter Analysis software. All data points were derived from MicroCal PEAQ-ITC (Malvern Panalytical) measurements collected from previous studies (2–4). R^2^ values reflect the square of Pearson’s correlation coefficient from linear regression; no R^2^ value or fitted parameter error was computed for the β-1,4-linked xylo-oligosaccharides data set, as it consists of only two data points.

Equation 1 shows the empirical linear relationship commonly used to analyze apparent EEC in protein-ligand binding:


(1) $$\begin{array}{c} \Delta H=T_{c}\Delta S+\beta \; \end{array}$$

where *T*_*c*_ is the compensation temperature, and β represents the intercept. We applied this approach to two recently described maltodextrin-binding proteins, Athe_2310 and Athe_2574, which display orthogonal substrate-length preferences (3). Both proteins bind α-linked glucans, but Athe_2310 preferentially recognizes shorter substrates (e.g., maltose or maltotriose), and Athe_2574 binds longer ligands (e.g., maltotetraose or maltoheptaose). Figure 1A shows that both maltodextrin-binding proteins exhibit trends consistent with length-dependent EEC behavior.

We next asked whether a multifunctional SBP accesses distinct thermodynamic regimes for different ligand classes. We examined Athe_2052, which we have proposed to be an SBP specific to branched xyloglucan oligosaccharides while possessing promiscuous binding affinity for other unbranched β−1,4-linked oligosaccharides (4). Figure 1B shows linear regression parameters comparable across cello-oligosaccharides (*T*_*c*_ = 247 ± 92 K and β = −8.8 ± 0.8 kcal/mol) and xylo-oligosaccharides (*T*_*c*_ = 226 K and β = −7.2 kcal/mol), suggesting that the Athe_2052 binding pocket retains a similar thermodynamic environment across both ligand classes.

Although both Athe_0597 and Athe_2052 recognize cello-oligosaccharides of similar lengths, Athe_0597 shows greater variability in enthalpic contributions (CV_Δ*H*_ > CV_Δ*S*_), whereas Athe_2052 shows greater variability in entropic contributions (CV_Δ*S*_ > CV_Δ*H*_) (Table 1). This suggests that different SBPs employ distinct thermodynamic partitioning even for the same ligand class, and that EEC is not solely ligand-driven.

**TABLE 1:** Summary of thermodynamic variation and fitted EEC parameters for each SBP and ligand class^*a*^

SBP	Ligand class	CV_Δ*H*_	CV_Δ*S*_	CV_Δ*G*_	Compensation temperature *T*_*c*_ (K)	Intercept (kcal/mol)
Athe_0597	Cello-oligosaccharides (G2–G5)	0.259	0.056	0.066	455 ± 122	−15.0 ± 5.2
Athe_2310	Malto-oligosaccharides (G2–G3) and trehalose (G2)	0.199	0.115	0.029	333 ± 114	−7.9 ± 1.7
Athe_2574	Malto-oligosaccharides (G4–G7)	0.224	1.211	0.039	411 ± 35	−10.8 ± 0.3
Athe_2052	Xylo-oligosaccharides (X4–X5)	0.180	0.357	0.034	226^*b*^	−7.2^*b*^
	Cello-oligosaccharides (G3–G5)	0.100	0.283	0.029	247 ± 92	−8.8 ± 0.8

^*a*^ Shown here are the coefficients of variation (CV) computed for enthalpy change (∆*H*), entropy change (∆*S*), and Gibbs free energy change of binding (∆*G*), as well as the compensation temperature and intercept obtained from linear regression using equation 1. Errors reflect the standard deviation errors in the calculation of the fitted regression parameters.

^*b*^ Since only two data points are present, the standard deviation error in the slope and y-intercept could not be calculated.

These comparisons are informative, but their mechanistic interpretation warrants caution. Sharp (1999) noted that ostensible EEC behavior can arise as an experimental artifact, particularly when ∆*H* and ∆*G* measurement errors are substantial (5). The thermodynamic potentials *G* and *H* are interrelated via Maxwell’s relations; hence, a linear relationship between ∆*H* and ∆*S* can arise trivially whenever ∆*H* variation exceeds ∆*G* variation, without necessarily implying any extra-thermodynamic behavior. Krug et al. (6) proposed a condition (equation 2) to determine whether observed EEC compensation patterns may simply reflect an artifact:


(2) $$\begin{array}{c} T_{c}-2\sigma <T<T_{c}+2\sigma \end{array}$$

where *T* represents the experimental temperature and σ represents the standard error of *T*_*c*_ from the fit (6). At *T* = 298 K, the fits for Athe_0597, Athe_2052, and Athe_2310 satisfy the Krug criterion, while Athe_2574 does not, suggesting that apparent EEC behavior in our data sets should be interpreted conservatively.

However, EEC has been recognized as important in biomolecular recognition, and its analysis remains useful as a comparative framework for generating hypotheses across SBP-carbohydrate systems (7). Our findings suggest three possibilities: (i) differences in substrate length preference may give rise to distinct thermodynamic compensation parameters; (ii) comparable thermodynamic compensation behavior may be retained across different ligand classes; (iii) the same ligand class, e.g., cello-oligosaccharides, may access distinct thermodynamic regimes when bound by different SBPs. These observations raise the possibility that compensation temperature and fitted intercept reflect intrinsic properties of the local binding environment and conformational landscape of a given SBP. Apparent EEC behavior may serve as a useful comparative descriptor of SBP-carbohydrate recognition, while underscoring the need for deeper structural and biochemical analysis to genuinely elucidate the mechanism.
